# Effect of (poly)phenol-rich ‘Daux Belan’ apple supplementation on diet-induced obesity and glucose intolerance in C57BL/6NCrl mice

**DOI:** 10.1038/s41598-023-43687-6

**Published:** 2023-10-11

**Authors:** Cindy H. J. Yu, Petra C. Kienesberger, Thomas Pulinilkunnil, H. P. Vasantha Rupasinghe

**Affiliations:** 1 Department of Plant, Food, and Environmental Sciences, Faculty of Agriculture, Dalhousie University, Truro, NS B2N 5E3 Canada; 2 Department of Biochemistry and Molecular Biology, Faculty of Medicine, Dalhousie University, Dalhousie Medicine New Brunswick, Saint John, NB Canada; 3 Department of Pathology, Faculty of Medicine, Dalhousie University, Halifax, NS Canada

**Keywords:** Biochemistry, Diseases

## Abstract

Obesity is a state of metabolic dysfunction that can lead to dyslipidemia and impaired glucose homeostasis. Apple polyphenols have been shown to ameliorate dyslipidemia/metabolic dysfunction in humans. The influence of apple (poly)phenols on energy metabolism in high-fat (HF) diet-induced obese mice remains controversial. This study examined the effect of dietary supplementation of (poly)phenol-rich ‘Daux Belan’ apple (DB; 6.2 mg gallic acid equivalence (GAE)/mouse/day; 0.15% (poly)phenol) in the form of freeze-dried powder on glucose and lipid metabolism in male HF-fed C57BL/6NCrl mice, in comparison to low-(poly)phenol-containing ‘Zestar’ apple (Z; 0.4 mg GAE/mouse/day). Obesity, glucose intolerance, hypertriglyceridemia, and hepatic lipid vacuolation were induced by HF feeding while circulating cholesterol levels remained unchanged. DB apple supplementation did not protect against HF-induced body weight gain, hyperglycemia, hepatic triglyceride level elevation, and hepatic lipid vacuolation at the tested dosage. Future studies should be conducted with increased DB dosage and employ apple (poly)phenols supplemented in the form of extracts or sugar-free powder.

## Introduction

The global prevalence of metabolic disorders such as type 2 diabetes mellitus (T2D) is increasing^1^. T2D is a multifactorial chronic disorder that disrupts carbohydrate, protein, and lipid metabolism. The combination of obesity and obesity-triggered insulin resistance strongly contributes to the development of T2D^2^. T2D is characterized by prolonged hyperglycemia that is caused by a wide array of metabolic imbalances, including insufficient insulin secretion by pancreatic β-cells, impaired glucose uptake due to insulin resistance in peripheral tissues, enhanced glycogenolysis, upregulated gluconeogenesis, and altered insulin-signalling pathway in insulin target tissues^3^. Lifestyle modifications such as increased physical activity, reduced body weight, increased intake of dietary fiber, whole grains, fruits and vegetables, as well as reduced saturated fat consumption are protective against progression into T2D in patients with slightly higher than normal blood glucose levels^4^.

Apple is a popular fruit among consumers for its availability and affordability. It is also a good source of dietary (poly)phenols^5^. In vitro blood glucose lowering action by apple (poly)phenols is well-documented, particularly in inhibiting carbohydrate-hydrolyzing enzymes, such as α-glucosidase and α-amylase enzymes^6,7^, that metabolize polysaccharides into glucose as well as inhibiting glucose transporter proteins that facilitate glucose uptake, such as SGLT-1 and GLUTs^8^. In various strains of mice and rats, apple (poly)phenols improved high fat (HF)-induced body weight gain^9,10^, blood glucose dysregulation^10,11^, and hyperlipidemia^9^.

Doses of 600 and 1200 mg total apple (poly)phenol/day induced a reduction in blood glucose in pre-diabetic people and healthy people, respectively^12,13^. In obese subjects, (poly)phenol-rich diets (2861 and 2903 mg/day) improved glucose regulation and insulin sensitivity^14^. However, studies have also reported no association with protection from impaired glucose homeostasis at total dietary (poly)phenol doses of 1200 mg/day or higher^15^. The imperfect concordance of the recommended daily (poly)phenol intake for T2D prevention and management in the literature complicates the establishment of a reference intake amount, likely due to heterogeneity of the studies and limitations in the estimation of (poly)phenol intake^15^. Thus, based on the evidence in the literature, the therapeutic dose was set at 1200 mg total apple (poly)phenol/day/human in this work, translating to 6.2 mg gallic acid equivalence (GAE)/mouse/day at the diet dose of 0.15% apple (poly)phenol. The in vitro anti-diabetic properties of (poly)phenol-rich ‘Daux Belan’ (DB) apples have been demonstrated in our previous work^7^. Therefore, in this study, the effect of DB whole apple powder supplementation on glucose and lipid homeostasis was investigated using a diet-induced obese and glucose-intolerant mouse model.

## Results

### Effect of apple supplementation on food intake, (poly)phenol intake, and body weight

**Figure 1:**
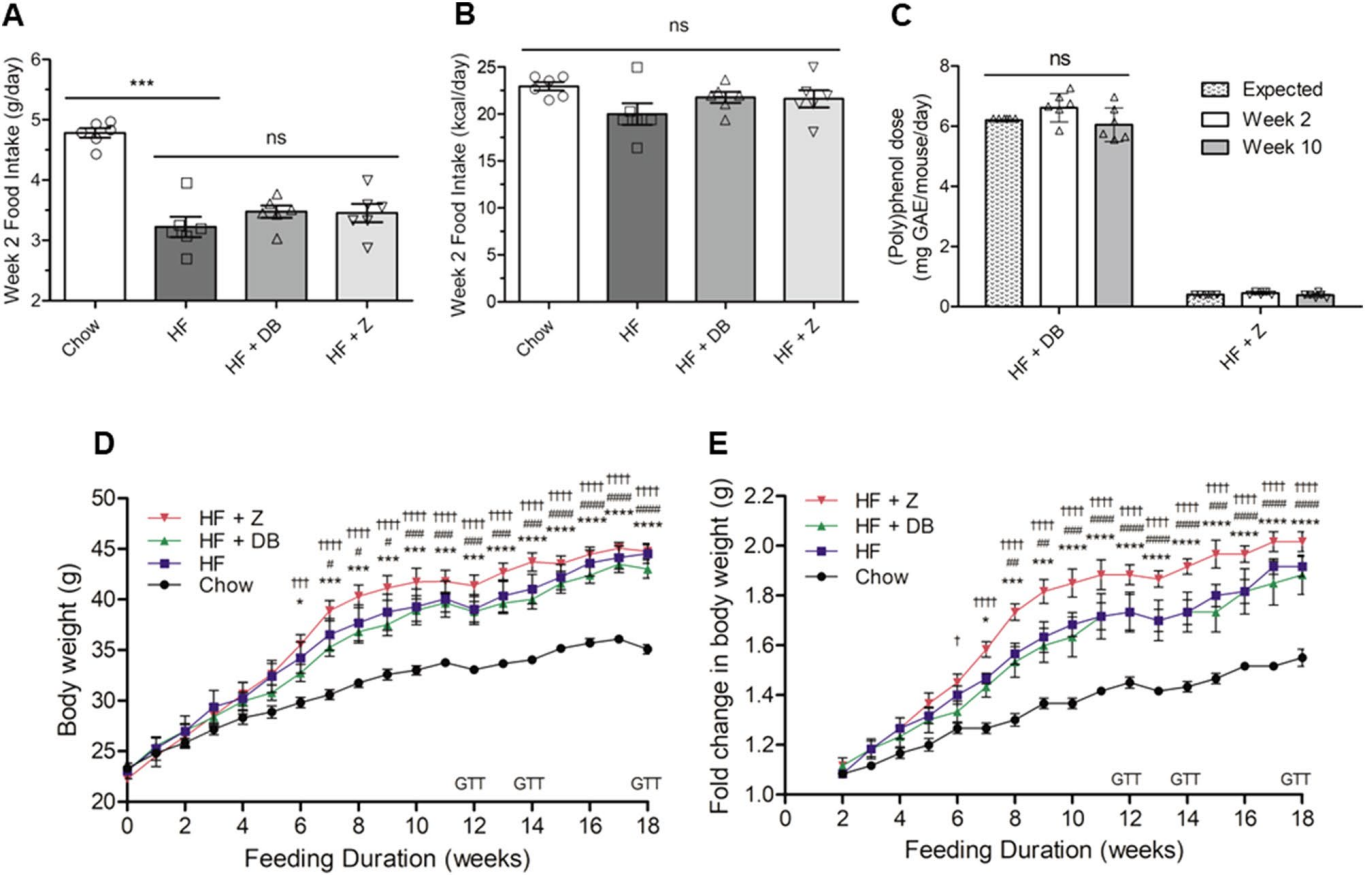
Effects of (poly)phenol-rich ‘Daux Belan’ apple powder supplementation on food intake and body weight in high-fat-fed male C57BL/6 mice. (**A**) Food intake (g/day). (**B**) Calorie intake (kcal/day). Calorie intake was calculated from food intake using the following calculator: https://www.omnicalculator.com/conversion/grams-to-calories. (**C**) (Poly)phenol dosage was measured at week 2 and week 10. Expected daily (poly)phenol dosage was 6.2 mg gallic acid equivalence (GAE)/mouse/day and 0.4 mg GAE/mouse/day for DB and Z groups, respectively. (**D**) Body weight. (**E**) Body weight gain is expressed as a fold change in starting body weight. (Fold change = final weight/initial weight). For A-E, n = 6. Statistical analyses were performed using one-way ANOVA followed by Tukey’s multiple comparisons tests (α = 0.05) for food intake and (poly)phenol dosage. Two-way repeated measures ANOVA followed by Tukey’s multiple comparisons test (α = 0.05) was performed for body weight analyses. Significance codes were assigned as followed: *P < 0.05, **P < 0.01, ***P < 0.001, ****P < 0.0001 for chow vs HF; ^#^P < 0.05, ^##^P < 0.01, ^###^P < 0.001, ^####^P < 0.0001 for chow vs HF + DB; ^†^P < 0.05, ^††^P < 0.01, ^†††^P < 0.001, ^††††^P < 0.0001 for chow vs HF + Z. *NS* not significant.

Food intake data collected at week 2 (Fig. 1A) showed that HF-fed mice consumed significantly less food compared with chow-fed mice (P < 0.001), while calorie intake (Fig. 1B) across all groups remain similar (P > 0.05). Neither (poly)phenol-rich DB nor low-(poly)phenol Z supplementation influenced food intake in the HF groups, indicating that apple powder supplementation did not interfere with feed palatability. At week 2, the mean daily (poly)phenol consumption was 6.6 ± 0.47 mg GAE/mouse/day for DB and 0.5 ± 0.05 mg GAE/mouse/day for Z (Fig. 1C). At week 10, the mean daily (poly)phenol consumption was 6.0 ± 0.59 and 0.4 ± 0.07 mg GAE/mouse/day for DB and Z, respectively. Despite variability between mice within diets, the difference in means of (poly)phenol intake was not significant (P > 0.05) from the expected DB (6.2 mg GAE/mouse/day; 0.15% diet) and Z (0.4 mg GAE/mouse/day) dosage at both week 2 and week 10 (Fig. 1C), meaning that the amount of apple (poly)phenols the mice consumed were consistent with the expectation. Obesity was induced in HF and HF + Z groups starting at week 6, and in HF + DB group at 7 weeks, signified by sharp increases in body weights compared to the chow group (Fig. 1D,E). Apple powder supplementation did not protect against HF-induced body weight gain.

### Effects of apple supplementation on glucose tolerance

**Figure 2:**
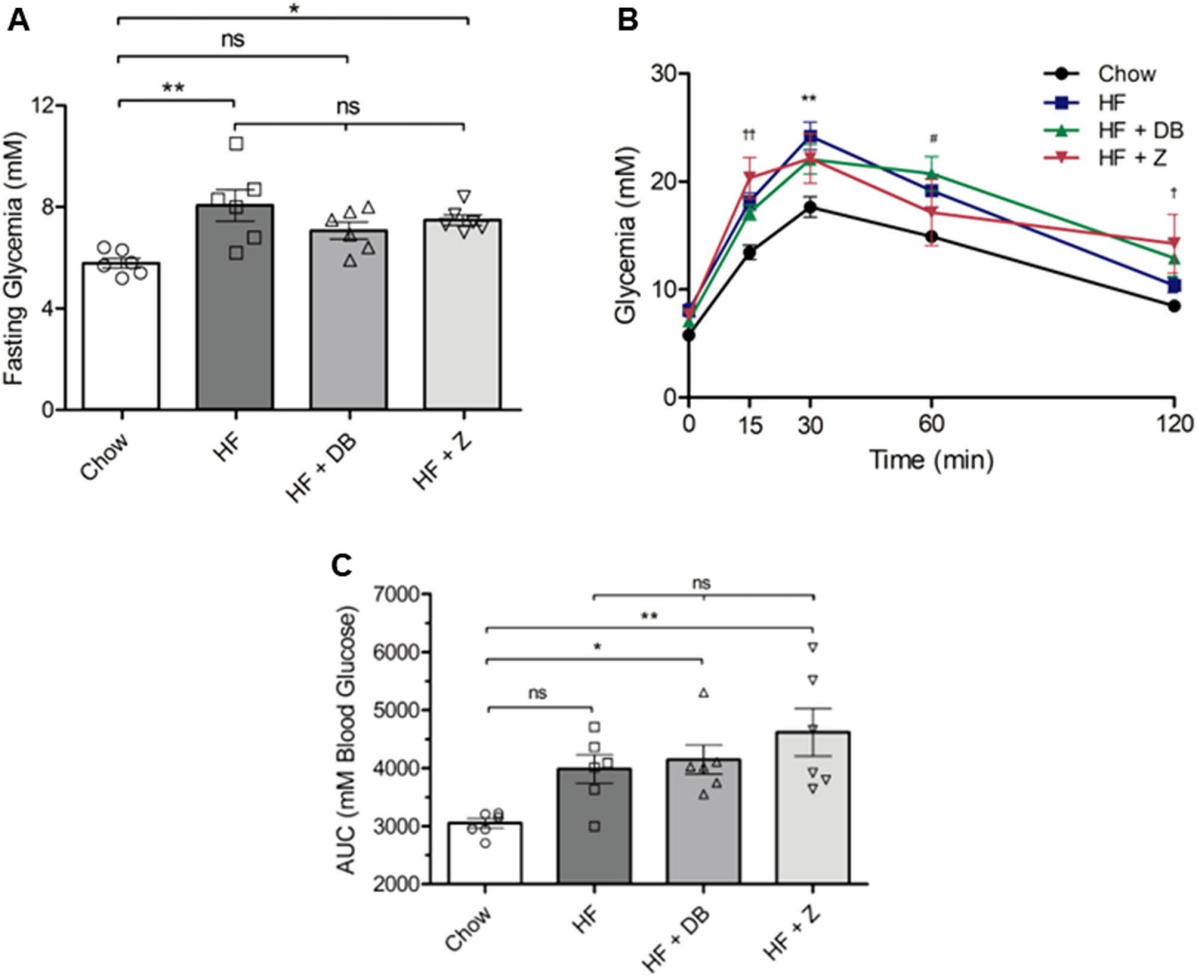
Effects of (poly)phenol-rich ‘Daux Belan’ apple powder supplementation on glucose homeostasis in high-fat-fed male C57BL/6 mice. (**A**) Fasting blood glucose at week 18, (**B**) glucose tolerance test at week 18, and (**C**) associated area under the curve. For (**A**–**C**), n = 6. Statistical analyses were performed using one-way ANOVA followed by Tukey’s multiple comparisons tests (α = 0.05) for all, except for glucose tolerance test at week 18, which was analyzed using two-way repeated measures ANOVA followed by Bonferroni post test (α = 0.05). Significance codes were assigned as followed: *P < 0.05, **P < 0.01, ***P < 0.001 for chow vs HF; ^#^*P* < 0.05, ^##^*P* < 0.01, ^###^*P* < 0.001 for chow vs HF + DB; ^†^*P* < 0.05, ^††^*P* < 0.01, ^†††^*P* < 0.001 for chow vs HF + Z. *NS* not significant.

At week 18, HF-fed groups displayed elevated fasting blood glucose levels (Fig. 2A) and increased glycemic response to IPGTT (Fig. 2B,C) compared to the chow-fed group. Fasting glycemia, as well as glucose levels and area under the curve (AUC) during IPGTT were comparable between all HF groups, demonstrating that apple powder supplementation did not influence HF-induced changes in glucose homeostasis.

### Effects of apple supplementation on plasma and hepatic lipid parameters and liver weight

**Figure 3:**
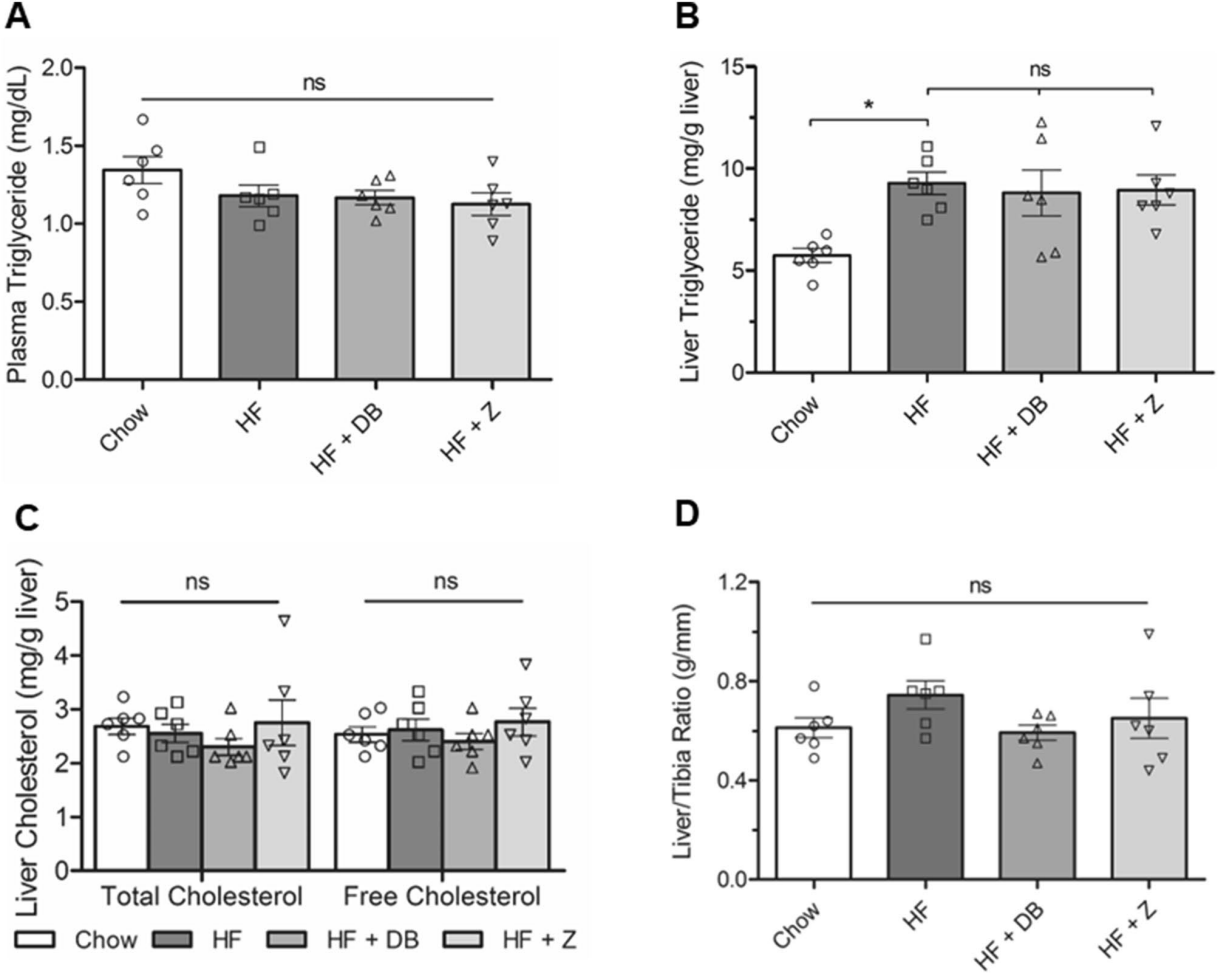
Effect of (poly)phenol-rich ‘Daux Belan’ apple powder supplementation on (**A**) plasma triglyceride, (**B**) liver triglyceride, (**C**) liver total and free cholesterol content, and (**D**) liver/tibia ratio (g/mm) in high-fat-fed male C57BL/6 mice. Statistical analyses were performed using one-way ANOVA followed by Tukey’s multiple comparisons tests (α = 0.05). Significance codes were assigned as followed: *P < 0.05, **P < 0.01, ***P < 0.001. *NS* not significant.

Plasma TG levels were comparable across all four diet groups (Fig. 3A). Liver TG content increased 1.6-fold in the HF group but was comparable between HF, HF + DB, and HF + Z groups (Fig. 3B). HF diet did not increase liver cholesterol levels (Fig. 3C) and total and free liver cholesterol levels were not altered by DB nor Z supplementation in the HF diet (*P* > 0.05). Similarly, neither the HF diet nor the apple powder supplementation impacted liver weights across all diet groups (Fig. 3D). Apple powder supplementation did not protect against HF-induced hepatic triglyceride elevation.

### Effects of apple supplementation on liver histopathology

**Figure 4:**
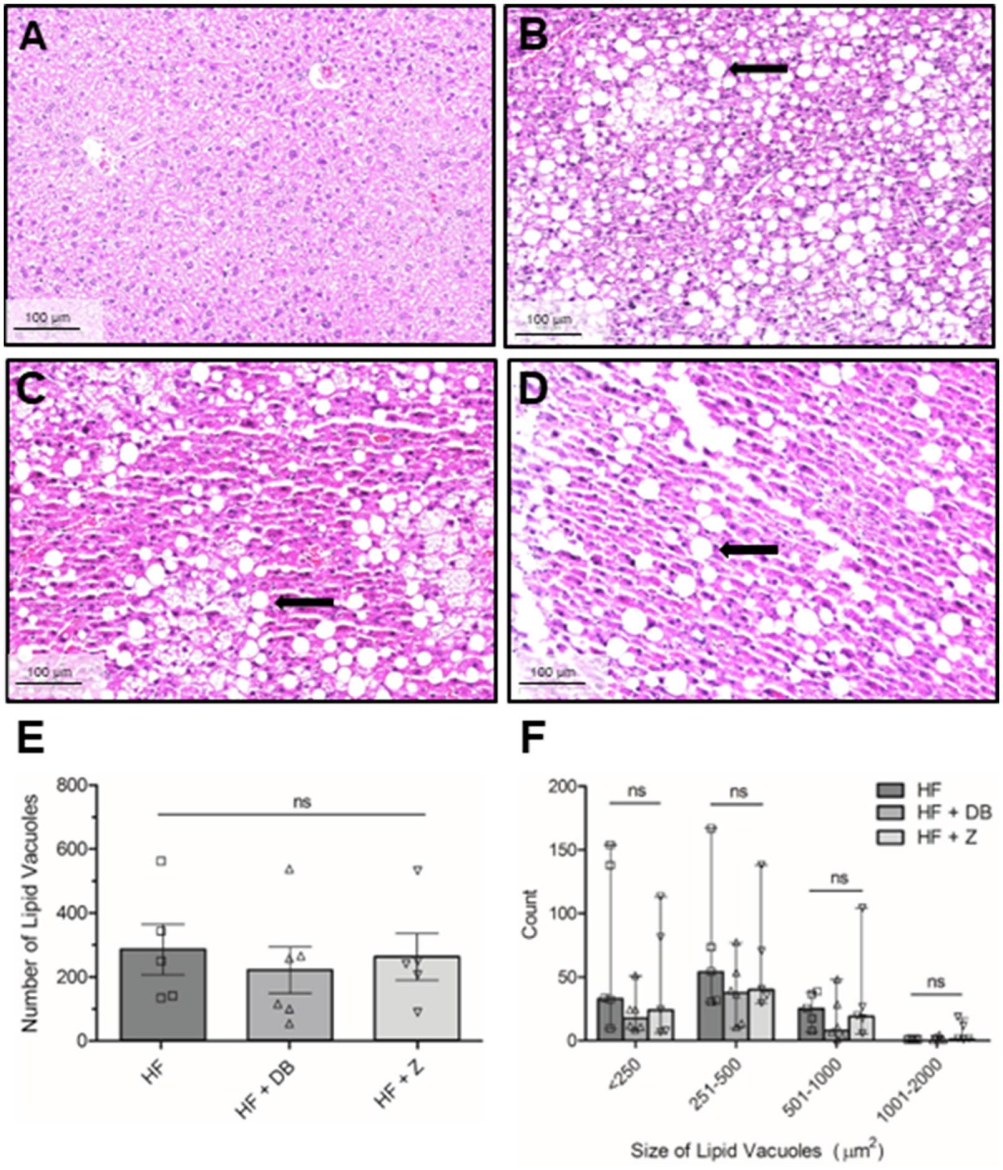
Liver histopathology examination (H&E staining) of hepatic lipid vacuolation in male C57BL/6 mice. (**A**) Chow, (**B**) high fat diet, (**C**) high fat + (poly)phenol-rich ‘Daux Belan’ apple powder, and (**D**) high fat + low (poly)phenol ‘Zestar’ apple powder. Round vacuoles indicated by the black arrow represent lipid deposition in high-fat-fed mice that were washed out during tissue processing. The number of liver lipid vacuoles (**E**) and size distribution of liver lipid vacuoles (**F**) in high-fat-fed male C57BL/6 mice. Statistical analyses were performed using one-way ANOVA followed by Tukey’s multiple comparisons tests (α = 0.05). Scale bar = 100 µm; 20 × magnification). *NS* not significant.

Chow-fed mice showed round central hepatocyte nuclei with no lipid vacuolation (Fig. 4A). HF-fed mice developed significant hepatic lipid vacuolation which compressed hepatocytes and their nuclei (Fig. 4B). Apple powder supplementation in the HF diet did not significantly repress hepatic lipid vacuolation (Fig. 4C,D). The number and size of liver lipid vacuoles (Fig. 4E,F) were similar across all HF groups (*P* > 0.05). Apple powder supplementation did not protect against HF-induced hepatic lipid vacuolation.

## Discussion

There is limited scientific literature that explored the effect of apple (poly)phenols on glucose and lipid metabolism in HF-induced obese C57BL/6 mice. Only apple (poly)phenol extracts^9,10,16^ and cloudy apple juice^17^ have been studied. This work is the first to evaluate apple (poly)phenol supplementation in whole-fruit powder form. The supplementation of apples in the whole fruit form (peel and flesh without the core) is advantageous because, in addition to providing (poly)phenols, apples contain other valuable bioactive constituents with a positive effect on glucose homeostasis such as insoluble fibre^18^ and pectin^19^, and consumption of dietary fiber also promotes satiety^20^.

As expected, HF feeding induced obesity in mice (Fig. 1B), however, apple powder supplementation did not affect body weight gain. Total (poly)phenol content (TPC) decreases as the fruit matures, meaning unripe apples are higher in TPC than ripe apples^21^. It is conceivable that the usage of unripe fruit (poly)phenols rather than mature whole fruit influences body weight gain. Another possible reason for not observing the significant impact of DB supplementation on the measured parameters is the dosage of apple (poly)phenols employed. This work is the first study to explore whole apple powder supplementation in a diet-induced obesity (DIO) model using C57BL/6 mice. The DB dose of 62 mg GAE/mouse/day (a 0.15% apple (poly)phenol), which is equivalent to 1200 mg GAE/adult/day, was employed in this study. Both 600 and 1200 mg total apple (poly)phenol/day are reported effective hypoglycemic doses in borderline diabetic people and healthy people, respectively^12,13^. Despite the use of allometric scaling in dose extrapolation from human to mouse in this study, where the human dose is extrapolated to an animal equivalent dose based on the body surface area normalization method^22^, the positive outcomes observed in reported human studies were not replicated in the animals in this study. The effect of (poly)phenols on glucose homeostasis could be different based on the nature of (poly)phenol sources such as whole fruit powder versus extract^23^. Further, intact (poly)phenols supplemented by whole apples that were not absorbed in the small intestines require biotransformation into metabolites for utilization by gut microbiota in the colon^24^. It is reported that mice contain different gut bacteria genera and species than humans^25^, suggesting potential differences in (poly)phenol metabolism between mice and humans and that it is possible that mice are not a very good model system for (poly)phenol metabolism. However, limited scientific literature report anti-obesity effects of 0.5%^10^ and 5%^16^ unripe Fuji apple (poly)phenol extract supplementation in the DIO model of C57BL/6 mice, but not 5% apple/kale extract^11^. As such, DB dose can be increased in future works to investigate potential weight gain and glycemia protective actions as suggested by in vitro findings in the literature. Although fasting glucose and glucose tolerance were comparable among HF diet-fed groups, it is possible that plasma insulin levels and systemic insulin sensitivity are altered by apple powder supplementation, which should be investigated in future studies.

Impaired fasting glucose and impaired glucose intolerance increase the risk of T2D, and elevated TG and cholesterol are important indicators of obesity^26^. One widely-observed consequence of obesity is the development of non-alcoholic fatty liver disease, which results in increased liver weight^27^. It is possible that the animals in this study were insensitive to dyslipidemia, as indicated by the lack of elevation in hepatic cholesterol levels and liver weight induced by HF feeding over 18 weeks. Using the same DIO model, improved glucose tolerance^10,11,16^ and decreased serum TG and cholesterol levels by apple (poly)phenol^9,10^ has been documented. However, some are reporting decreased blood TG but not cholesterol levels by apple (poly)phenols^17,28^. Dose-dependent reduction in serum cholesterol and TG by apple (poly)phenol is observed using Kunming mice^29^. Here, we report that DB supplementation at a 0.15% diet dose did not influence obesity, and glucose and lipid homeostasis in HF-fed male C57BL/6 mice.

This study is subjected to several limitations that could be addressed in future research. Although inbred C57BL/6 mice are susceptible to DIO and are widely used in obesity research due to their identical genetics, they still display significant inter-individual variability in susceptibility to DIO and related phenotypes due to variability in HF diet consumption^30^. Specifically, they have been observed to display large variations in the initial basal fat mass, which is a significant predictor of body weight gain when later introduced to HF feeding^31^. In addition, susceptibility to DIO in C57BL/6 mice can also be influenced by various circumstances in the intra-uterus environment, early post-natal stage, and adulthood^30^. In this study, the lack of differences in lipid profile in HF-fed compared to chow-fed mice suggests possible insensitivity to DIO-induced dyslipidemia in this cohort of animals. As such, considering these factors that influence DIO susceptibility, potential counteracting approaches can include repeating the study in multiple cohorts of animals to deliver greater insights and distinguishing between HF-tolerant and intolerant animals, which can be accomplished by increasing the number of animals to offset variability while maintaining statistical power or by performing a median-based split on food intake data^32^. Additional blood parameters (total cholesterol and total protein)^33^, hepatic health parameters (aspartate aminotransferase and alanine aminotransferase activity)^33^, and organ weights (kidney and fat pad)^34^ can be determined in future studies to increase resolution of the metabolic impact of HF-feeding and apple powder supplementation. Moreover, whole apple supplementation has been found to reduce flavan-3-ols bioaccessibility and thus its serum concentration in minipigs when compared with extract supplementation^35^. Therefore, future studies are warranted to investigate the possible interaction among (poly)phenols and other constituents of whole apple powder. In the current study, total dietary (poly)phenol doses represented a human dose of 1200 mg GAE/day/adult; however, in future studies, higher doses i.e. 2500 to 3000 mg GAE/day/adult can be assessed in a dose–response manor. Strategies to enhance the bioaccessibility and bioavailability of (poly)phenol compounds in whole apple powder can be considered to improve diet intervention outcomes^36^. Lastly, apple (poly)phenol supplementation in the extract form, as sugar-free powders, or in combination with other fruits, vegetables or bioactive peptides/proteins are also potential areas to explore.

## Conclusion

(Poly)phenol-rich DB apple supplementation in the whole fruit form as freeze-dried powder did not attenuate HF-induced body weight gain, glucose intolerance, hepatic hypertriglyceridemia, and hepatic lipid vacuolation at the dosage of 6.2 mg GAE/mouse/day (at 0.15% diet). Suggestions for future studies include utilizing human cell models to observe dose-dependent changes in lipid parameters, testing higher dosages of apple powder in vivo, (poly)phenol extracts alone or in combination with other fruits, vegetables, or other bioactives over a shorter study period, studying the effects of sugar-free apple powder supplementation, and employing a different food matrix to improve the bioaccessibility of apple (poly)phenols. In conclusion, the potential use of apple (poly)phenols as value-added food and nutraceutical ingredients against glucose intolerance and adiposity warrants further investigations using well-designed cell and pre-clinical experimental animal models before recommendations on the T2D protective effects of apple (poly)phenols for subsequent studies involving human participants can be made.

## Materials and methods

### Animals

**Figure 5:**
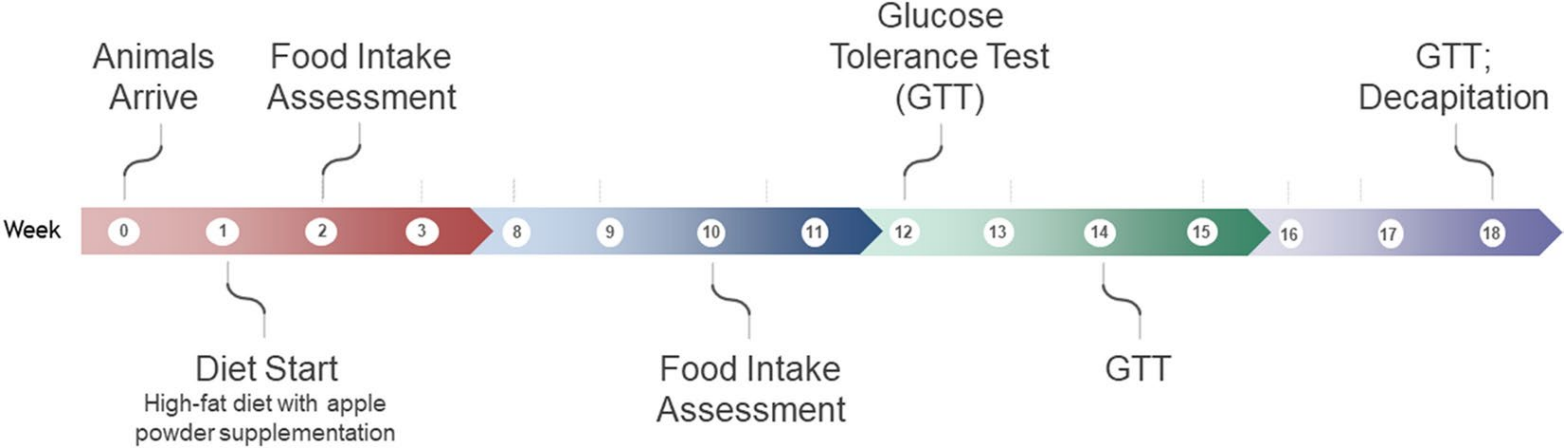
Timeline of procedures for the mouse study.

Twenty-four male mice C57BL/6NCrl (8 weeks old; Charles River Laboratories, Montreal, QC, Canada) were singly housed in a controlled environment (12 h day/night cycle, lights on between 7:00 to 19:00) with ad libitum access to food and water at Carleton Animal Care Facility of Dalhousie University, Halifax, NS, Canada. The animals were housed in a individual ventilated cages (IVC) rack system with a cage size of 11.75 inch length × 7.5 inch width × 5 inch height (Allentown™, Allentown, NJ, USA) and the room temperature of 21.2 °C and the average humidity of 20–21%. Following 1 week of adaptation to the chow diet, the mice were randomly divided and subjected to the following four diets (n = 6) for 18 weeks: (1) chow, (2) high fat (HF), (3) HF supplemented with (poly)phenol-rich ‘Daux Belan’ (DB) apple powder (HF + DB), and (4) HF supplemented with low-(poly)phenol-containing ‘Zestar’ (Z) apple powder (HF + Z). The design and protocols for the animal experiments were approved by the Dalhousie University Committee on Laboratory Animals (UCLA; Protocol # 21-005). All the experiments were performed in accordance with relevant guidelines and regulations of UCLA. The findings of the study were reported in accordance with Animal Research: Reporting of In Vivo Experiments (ARRIVE) guidelines. The timeline of procedures is outlined in Fig. 5.

### Preparation and compositional analysis of apple powders

The apples were harvested from the Apple Biodiversity Collection (Agriculture and Agri-Food Canada [AAFC] Research and Development Centre, Kentville, NS, Canada; 45°04′08″ N 64°28′41″ W) at appropriate maturity based on previously published data^37^. The apples were collected with the approval from AAFC. The collection of apples and the performance of experimental research on these two apple cultivars complied with the national guidelines of Canada. The taxonomical identification of the two cultivars was performed by a pomologist at AAFC. Six days following respective harvest dates, apples stored at 4 °C were cored, sliced, and immediately flash-frozen using liquid nitrogen. Frozen apple slices were freeze-dried (Dura-Stop Digital Control Stoppering Tray Dryer with Dura-Dry MP Microprocessor Control Corrosion Resistant Freeze-Dryer, FTS Systems™; Marbletown, NY, USA) at − 40 °C for 2 days, then at 10 °C for additional 2 days. Freeze-dried apple slices were ground into a fine powder using a coffee grinder (particle size approximately 10–100 µm) and stored at − 80 °C in sealed Ziploc bags until the commencement of the feeding trial. Freeze-dried DB and Z samples were analyzed for nutritional composition, where calories (by calculation), moisture (drying under vacuum method; AOAC 950.46b), protein (block digestion method; AOAC 981.10), fat (acid hydrolysis method; AOAC 922.06), ash (direct method; AOAC 920.153) and carbohydrate content (by calculation) were quantified. (Poly)phenolic compositions of DB and Z were characterized by the LC–ESI–MS^38^ (Table 1).

**Table 1:** Proximate and (poly)phenol composition of freeze-dried ‘Daux Belan’ and ‘Zestar’ apple powder.

Composition	‘Daux Belan’	‘Zestar’
Proximate composition (%)		
Moisture	7.4	10
Protein	2.0	1.7
Fat	2.0	1.7
Ash	1.4	1.5
Carbohydrates	87	85
Total sugars	67	69
Glucose	15	4.6
Fructose	36	36
Lactose	< 0.4	< 0.4
Sucrose	16	28
Maltose	< 0.4	< 0.4
Calories (kcal)	375	363
(Poly)phenol composition (µg/g fresh weight [FW])		
Chlorogenic acid	292 ± 22	282 ± 21
Cyanidin 3-galactoside	5.94 ± 0.34	176 ± 14
Quercetin glycosides	216 ± 24	74 ± 7.8
(−)-Epicatechin	291 ± 32	10.6 ± 1.73
d-(+)-Catechin	65 ± 6.3	0.73 ± 0.27
Phloridzin	119 ± 45	32 ± 3.6
Phloretin-2′-*O*-xyloglucoside	164 ± 5.3	73 ± 2.0
Procyanidin C1	341 ± 8.9	14.7 ± 1.7
Procyanidin B2	286 ± 6.5	115 ± 4.7
Total (poly)phenol content (µg/g FW)	2288	802

### Preparation of apple powder-incorporated HF diet

The average body weight of a human adult and an 8-week-old male C57BL/6 mouse were assumed to be 60 kg^22^ and 25 g^39^, respectively. The therapeutic dose determined for this study was 1200 mg gallic acid equivalents (GAE)/day/60 kg adult human, therefore the human dose for this study is 20 mg GAE/day/kg adult. Based on the human dose, the mice equivalent dose was calculated using Eq. (1), where the human and mouse Km factor (average body weight/body surface area) were 37 and 3, respectively^22^. As such, the animal (poly)phenol dose for this study was calculated to be 247 mg GAE/kg body weight mouse/day. The 0.15% diet dose of (poly)phenols was determined using the following equation: Diet dose (mg (poly)phenols/kg diet) = [single daily dose (mg (poly)phenols/kg BW/day) × body weight (g body weight/animal)]/daily food intake (g diet/day).

1 $${\text{Human}}\;{\text{dose}}\;({\text{mg/kg}}) = {\text{Animal}}\;{\text{dose}}\;({\text{mg/kg}}) \, \times \, \left( {{\text{Animal}}\;{\text{Km}}\;{\text{factor/Human}}\;{\text{Km}}\;{\text{factor}}} \right)$$

Freeze-dried DB (0.266 g dry weight/mouse/day) was incorporated into HF powder (Research Diet Inc., New Brunswick, NJ, USA; D12451; 45 kcal % fat) using a whisk in a clean metal bowl, and aseptic water was added as a binder. The mixture of apple powder and HF powder was then weighed and divided into equal portions by weight, formed into pellets by pressing with a measuring teaspoon. The pellets were then stored at 4 °C in a Ziploc bag for up to a week until feeding. With the assumption that a mouse consumes 4 g of food daily^40^, the HF + DB group and HF + Z group were receiving (poly)phenols from apple powders incorporated at equal weights at the approximate dosages of 6.2 mg GAE/mouse/day and 0.4 mg GAE/mouse/day, respectively. Apple powder weights were adjusted monthly according to body weight gain to maintain the apple (poly)phenol dosage supplemented. Percentages of calories provided by protein, carbohydrate, and fat in chow and HF diets were presented in Table 2. Detailed composition of chow (Lab Diet^®^, St. Louis, MO, USA; 5P04—Prolab RMH 3500; 15.5 kcal % fat) and HF diets are presented in Supplementary Table S1 and Fig. S1, respectively.

**Table 2:** Percentages of calories provided by protein, carbohydrate, and fat in chow and high-fat (HF) diets.

Calories provided by (%)	Chow diet	High-fat diet
Protein	26.2	20
Carbohydrate	58.3	35
Fat	15.5	45

### Body weight changes and feed intake analysis

Mice were weighed weekly. At week 2 and week 10, feed intake analysis was performed in all 24 single-housed animals for seven consecutive days. Fold change in body weight was calculated by dividing the final weight with the initial weight. On day 1, the weights of the feed hopper containing feed were obtained. From day 2 to 7, the weights of the feed hopper containing leftovers and the weights of the fresh feed given were monitored to calculate the amount of food consumed by the mice. Any feed dropped in the bedding was picked up and weighed. Calorie intake was obtained using a “gram to calorie calculator” (https://www.omnicalculator.com/conversion/grams-to-calories).

### Intraperitoneal glucose tolerance test (IPGTT)

IPGTT was performed as previously described^41^. At the beginning of weeks 12, 14, and 18, all mice (n = 24) were subjected to IPGTT. The body weights of the mice were recorded, then food was removed for a 16-h overnight fast with ad libitum water provided. Post-fast body weight was measured and used for glucose dosage calculation (2 g/kg). Pricking the tail vein with a needle and massaging the tail for a drop of blood, the basal blood glucose measurement was taken using a glucometer and glucose test strip (Accu-chek^®^ Guide, Roche Diabetes Care, Mississauga, ON, Canada). Thereafter, an IP injection of 20% glucose was administered and blood glucose concentrations were measured again at 15-, 30-, 60-, and 120-min post-injection.

### Animal euthanasia

At the end of the 18-week study (after completing the IPGTT), mice were euthanized by decapitation using a small animal guillotine (Braintree Scientific, Inc., MA, USA) following a four-hour fast. Due to the concern of anesthesia procedure exerting confounding effects on plasma metabolites^42^, anesthesia was not performed prior to decapitation in this study. Blood glucose concentration was determined using a glucometer (Accu-chek^®^ Guide, Roche Diabetes Care, Mississauga, ON, Canada). Whole blood was collected in EDTA-coated tubes (14.6% w/v EDTA) and spun at 2000×*g* for 10 min for the collection of plasma. A piece of the liver was placed in 10% formalin for histopathology analysis and the rest of the liver was flash-frozen in liquid nitrogen. Tibia was isolated and the length was measured using a ruler. Animal plasma and tissues were stored at − 80 °C until further analysis.

### Plasma and liver triglyceride content (TG), liver cholesterol contents and liver weight

Plasma TG (FUJIFILM Wako Shibayagi Corporation, Gunma, JP; 632-50991), liver TG (Cayman Chemical, MI, USA; 10010303), and liver total and free cholesterol contents (Abcam, Cambridge, UK; ab65359) were quantified using colorimetric assay kits according to the manufacturer's instructions. Liver weight was divided by tibia length to normalize for organ weight changes induced by disease^43^.

### Liver histopathology

Histologic analysis was performed based on a previously described method^44^. Fresh liver tissues were fixed in 10% formalin for 48 h at room temperature and stored in 70% alcohol at 4 °C until further processing. Fixed tissues were trimmed into appropriate sizes and shapes, embedded in paraffin wax, and sliced at 5 µm thickness. Hematoxylin and Eosin (H&E) staining of paraffin-embedded sections was performed as per commercial kit instructions (Abcam, Cambridge, UK; ab245880). Images of the liver slides were captured using the Pannoramic Midi Digital Slide Scanner (3DHISTECH Ltd., Budapest, Hungary). Visualization and enumeration of lipid vacuoles in the liver cross-sections were performed using SlideViewer software (3DHISTECH Ltd., Budapest, Hungary), while the area of lipid vacuoles in the liver cross-sections was analyzed in the ImageJ software (National Institute of Health, MD, USA) using Adiposoft plug-in.

### Statistical analysis

The plasma and liver samples of six animals from each treatment were assayed and all data were expressed as mean ± standard error of the mean (SEM). (Poly)phenol dosage results were expressed as mean ± standard deviation (SD). All statistical analyses were conducted using one-way ANOVA followed by Tukey’s multiple comparisons tests (⍺ = 0.05), with the exception of body weight and glycemia analyses, which were analyzed using two-way repeated measures ANOVA followed by Tukey’s multiple comparisons tests (⍺ = 0.05). The number of liver lipid vacuoles was expressed as median ± SEM and statistical analyses were carried out using the Kruskal–Wallis test. Data analyses and curation were performed in GraphPad Prism version 10 (GraphPad Software, San Diego, CA, USA).

### Ethics declarations

The design and protocols for the animal experiments were approved by the Dalhousie University Committee on Laboratory Animals (Protocol # 21-005).

### Supplementary Information

Supplementary Information.

## Data Availability

All data generated or analysed during this study are included in this published article (and its Supplementary Information files).
