# Supplementary material for: Effect of (poly)phenol-rich ‘Daux Belan’ apple supplementation on diet-induced obesity and glucose intolerance in C57BL/6NCrl mice
Source: Sci Rep. 2023 Oct 11;13:17206. doi: 10.1038/s41598-023-43687-6 (PMC10567707; doi:10.1038/s41598-023-43687-6)
Supplement: Supplementary file 1 — Supplementary Information. [file 41598_2023_43687_MOESM1_ESM.docx]

**Supplementary Materials**

Supplementary Figure S1. Detailed chow composition **
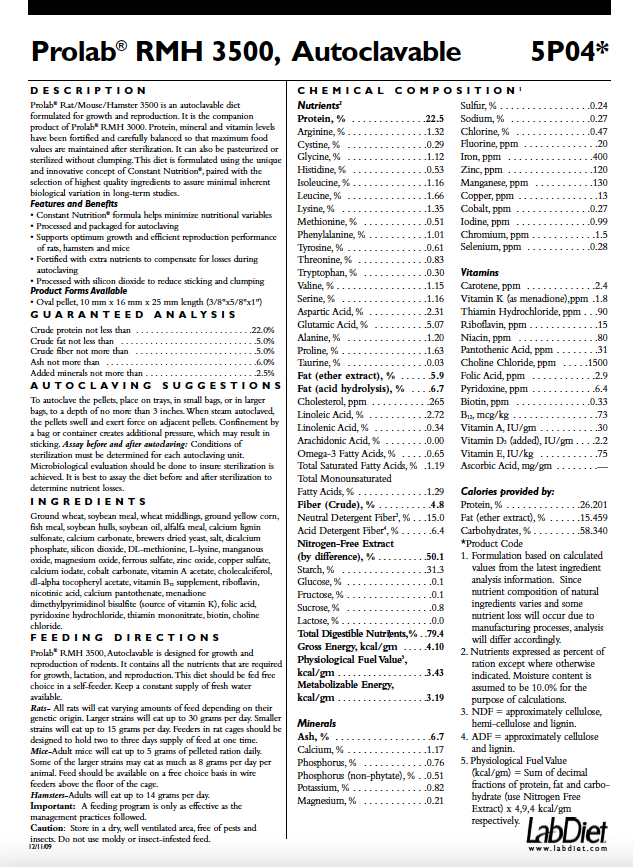
**

Supplementary Table S1. Detailed high-fat diet composition

| Ingredient | High-fat Diet (%) |
| --- | --- |
| Casein | 23.31 |
| L-Cystine | 0.35 |
| Sucrose | 20.60 |
| Lodex 10 | 11.65 |
| Corn Starch | 8.48 |
| Cellulose | 5.83 |
| Lard | 20.68 |
| Soybean Oil | 2.91 |
| Mineral Mix S10026B^a^ | 5.83 |
| Choline Bitartrate | 0.23 |
| Vitamin Mix V10001C^b^ | 0.12 |
| FD&C Red Dye #40 | 0.01 |

^a^ The mineral mix S10026B is composed of 17.98% sucrose, 33% potassium citrate, 26% calcium phosphate, 11% calcium carbonate, 5.18% sodium chloride, 5.15% magnesium sulfate, 0.84% magnesium oxide, 0.42% ferric citrate, 0.25% manganese carbonate, 0.11% zinc carbonate, 0.039% chromium potassium sulfate, 0.021% copper carbonate, 0.006% ammonium molybdate, 0.004% sodium fluoride, 0.001% sodium selenite, and 0.001% potassium iodate.

^b^ The vitamin mix V10001C is composed of 78.4% sucrose, 10% vitamin E, 3% niacin, 2% biotin, 1.6% pantothenic acid, 1% vitamin D3, 1% vitamin B12, 0.8% vitamin A, 0.7% pyridoxine HCl, 0.6% riboflavin, 0.6% thiamine HCl, 0.2% folic acid, and 0.1% menadione sodium bisulfite.

Note: Diet composition data obtained from Research Diets, Inc. https://www.researchdiets.com/formulas/d12451
